# Management of ADHD in children across Europe: patient demographics, physician characteristics and treatment patterns

**DOI:** 10.1007/s00431-013-1969-8

**Published:** 2013-02-26

**Authors:** Paul Hodgkins, Juliana Setyawan, Debanjali Mitra, Keith Davis, Javier Quintero, Moshe Fridman, Monica Shaw, Valerie Harpin

**Affiliations:** 1 Shire Development, LLC, 725 Chesterbrook Boulevard, Wayne, PA 19087 USA; 2 RTI Health Solutions, Research Triangle Park, NC USA; 3 Hospital Universitario Infanta Leonor, Madrid, Spain; 4 AMF Consulting, Inc, Los Angeles, CA USA; 5 Norgine Pharmaceuticals, Uxbridge, Middlesex UK; 6 Ryegate Children’s Centre, Sheffield, South Yorkshire UK

**Keywords:** Attention deficit/hyperactivity disorder, Methylphenidate, Amphetamine, Atomoxetine

## Abstract

This study was a retrospective chart review performed to examine and describe physician practice patterns in managing attention deficit/hyperactivity disorder (ADHD) across Europe. Physicians treating ADHD in the UK, France, Germany, Italy, the Netherlands and Spain were recruited. Each physician abstracted medical records of five patients (aged 6–17 years at time of review) with a documented diagnosis of ADHD made between January 2004 and June 2007. Data provided by the physician via the abstraction included (a) physician characteristics, (b) patient characteristics, (c) ADHD diagnosis and (d) ADHD outcomes (adherence, symptom control and satisfaction). A total of 779 patients met study inclusion criteria. In the overall population, patients’ mean (SD) age at time of diagnosis was 8.9 (2.6) years. The predominant treatment choice was long-acting methylphenidate, which was prescribed to more than 56 % of patients. According to physicians, only 30.8 % of patients showed ‘complete symptom control’ on current treatment and only 31.8 % of physicians reported being ‘very satisfied’ with their patients’ current treatment. Physicians’ assessments of complete symptom control and physician satisfaction with treatment were low, indicating unmet needs with current ADHD management in Europe.

## Introduction

Attention deficit/hyperactivity disorder (ADHD) affects as many as 3 to 9 % of children worldwide [15, 19] and is associated with significant and wide-ranging impairments. ADHD is one of the most common psychiatric disorders in childhood and is characterized by developmentally inappropriate levels of inattention, hyperactivity and/or impulsivity [2]. Children with ADHD struggle in the areas of academic functioning, self-esteem and interpersonal relationships [1, 13, 14], and they are at higher risk for mental health comorbidities such as mood disorders and substance use disorders [4, 5, 8, 22]. Studies have shown that families of children with ADHD experience considerable emotional and financial stressors [10, 12, 23], and ADHD has also been shown to negatively impact the health-related quality of life for both children and adults [9]. Taken together, these negative short- and long-term outcomes of ADHD on patients and families make it a public health concern and affirm the need for effective treatment [15].

According to European guidelines, the diagnosis and management of ADHD consists of nonpharmacological options, including behavioural therapy (BT), and pharmacological options, including stimulants (long- and short-acting methylphenidates and amphetamines) and nonstimulants [15, 24]. However, the availability of and access to treatments vary across countries. Although much is known about the efficacy and safety of individual ADHD treatment options via randomized, controlled clinical trials, real-world treatment and utilization patterns of current ADHD therapies as well as physician practice patterns in the management of ADHD across European countries have not been studied extensively. Cultural beliefs and differences across countries contribute to the real-world variation regarding how ADHD is managed. To better understand current ADHD management in ‘the real world’ across Europe, it is important to understand how physicians in different countries diagnose and manage ADHD. It is also important to understand physician’s perceptions on how currently available medications to treat ADHD are working to manage the condition. The purpose of this retrospective chart review was to descriptively illustrate variation in physician practice patterns in the management of ADHD in various Western European countries.

## Methods

**Fig. 1:**
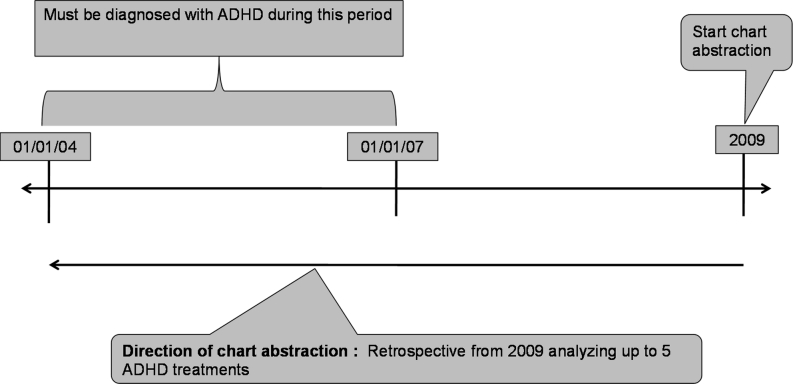
Study design

This study was designed as a retrospective review of patient medical records by their treating physicians (Fig. 1). In 2009, paediatricians, neuropaediatricians, child and/or adolescent psychiatrists and paediatric neurologists who treated patients with ADHD were identified from a database of healthcare providers in six countries in Western Europe: the UK, France, Germany, Italy, the Netherlands and Spain. Participating physicians were identified through the following sources: (a) physician directories maintained by local European medical associations and (b) local physician telephone directories. Overall, no formal sampling procedure was employed for physician recruitment; however, physicians with ADHD expertise or prior positive working relationships with the fieldwork company were initially selected to be contacted for study participation.

Physicians were contacted by either email or phone calls to assess physician eligibility and interest in participating in the study. If eligible physicians indicated interest, they were directed to complete a web-based screening questionnaire. Physicians were screened as eligible for inclusion in the study if they were engaged in clinical practice for between 3 and 30 years, managed the treatment of at least five ADHD patients (aged 6–17 years) per month and were responsible for making ADHD treatment decisions.

Physicians were required to identify the most recent ADHD patients (up to a maximum of five patients aged 6–17 years) that they had seen at the time of the review. In order for patients to be included in the study, they should have had a documented diagnosis of ADHD between January 2004 and June 2007 and have had at least 2 years of follow-up post-diagnosis. Patients were also required to have received either pharmacological treatment or BT following the ADHD diagnosis. Patient charts were excluded if there was evidence of enrolment in a randomized clinical trial. No other specific sampling strategies were employed.

Physicians abstracted patient chart data, entered the data into an electronic web-based form and then translated it into the local language. Instructions for completing the chart abstraction were provided on the web-based form. The data collection tool was designed in such a way that physicians were not able to skip any questions; however, if certain information was not available in the patients’ chart, physicians could select a ‘don’t know’ option. Wording for key questions regarding satisfaction, symptom control and adherence to the treatment regimen has been included in the Appendix. Each chart abstraction form was estimated to take 15 to 30 min to complete. All data gathered were de-identified and made completely anonymous. As no identifying patient information was collected, physicians did not have to obtain permission from patients before abstracting charts. Physicians were nominally compensated for their time.

### Variables for assessment

Both physician characteristics and patient characteristics were collected. The following patient characteristics were collected from the chart: age at diagnosis and at start of treatment, gender, ethnicity, insurance status, education level, reasons for seeking evaluation, comorbidities at diagnosis and the level of patient engagement and family involvement with the condition and its treatment on a ten-point scale where 1 represents ‘not engaged/involved at all’ and 10 represents ‘strongly engaged/involved’. Other ADHD-related information collected included ADHD diagnostic criteria utilized, ADHD treatment goals, ADHD symptom presentation and levels of impairment at the time of ADHD diagnosis on a ten-point scale where 1 represented ‘no impairment’ and 10 represented ‘high level of impairment’ (from 2004 to 2007). Also, treatment modalities were collected starting from the time of chart abstraction and going backwards up to five previous ADHD treatments. Data collected on treatment modalities included dose, prescription date and reason for changing dose and/or therapy. A switch from one treatment modality to another or a discontinuation of treatment was considered as new treatment. Dose changes for the same medication were not considered as new treatment. Data on treatments administered in other clinics or those that exceeded the last five treatments were not documented.

Data on adherence to current and previous ADHD treatments were also collected. Physicians were asked to report on the patient’s ADHD symptom control (‘completely’, ‘moderately’, ‘poorly’ or ‘not controlled’) and their satisfaction level (‘very satisfied’, ‘moderately satisfied’, ‘neither satisfied nor dissatisfied’, ‘moderately dissatisfied’ or ‘very dissatisfied’) with treatment at time of chart abstraction.

### Variable transformations

Associations between patient characteristics and outcomes were examined using a binary outcome definition for adherence, physician perception of symptom control and physician satisfaction (all with current therapy at time of review) to simplify the analysis and its interpretation. Physician-estimated adherence to treatment was defined as >80 % adherence during weekdays and >50 % adherence during weekends and holidays in accordance with the literature characterizing the medication possession ratio [20] and its use in assessing ADHD medication adherence [3]. The four levels of symptom control were dichotomized to ‘completely controlled’ vs ‘moderately’, ‘poorly’ or ‘not controlled’. The five levels of physician satisfaction were dichotomized to ‘very satisfied’ vs ‘moderately satisfied’, ‘neither satisfied nor dissatisfied’, ‘moderately dissatisfied’ or ‘very dissatisfied’.

### Statistical analysis

All study variables were summarized descriptively. Continuous variables were summarized and expressed in mean values, medians, ranges and standard deviations. Categorical variables were summarized in the form of proportion and frequency distributions. Summary statistics are provided by country for physician, patient and treatment characteristics and for diagnosis criteria used.

The significance (*p* < 0.05) of the associations was tested using Fisher’s exact or *t*-tests for dichotomous and continuous variables, respectively. Odds ratios were reported for dichotomous values and mean values were reported for continuous variables.

All data were analysed using statistical software (SAS Version 9, SAS Institute, Inc., Cary, NC, USA).

## Results

### Physician characteristics

#### Physician demographics

A total of 340 physicians reviewed and abstracted charts for 779 patients. On average, physicians were engaged in clinical practice for 15 years (range 3 to 30 years). Each physician managed approximately 20 patients aged 6 to 12 years and 15 patients aged 13 to 17 years. Medical specialties are listed in Table 1.

**Table 1:** ADHD diagnostic criteria and scales utilized by specialty for each country (physicians could select more than one diagnostic criterion utilized at diagnosis)

Country	Specialty (*n*)	Patients (*n*)	ADHD diagnostic criteria and scales, *n* (%)					
			DSM-IV	ICD-9/ICD-10	SNAP-IV	Connors	IOWA	Other
France	Paediatrician (1)	5	5 (100.0)	0	0	5 (100.0)	0	0
	Neuropaediatrician (2)	7	6 (85.7)	6 (85.7)	5 (71.4)	2 (28.6)	0	0
	Neuropsychiatrist (1)	2	2 (100)	0	0	0	0	0
	Psychiatrist (26)	60	39 (65.0)	31 (51.7)	7 (11.7)	25 (41.7)	1 (1.7)	0
	Paediatric/adolescent psychiatrist (18)	53	35 (66.0)	26 (49.1)	1 (1.9)	31 (58.5)	0	0
	Neurologist (2)	3	3 (100)	0	0	1 (33.3)	0	0
	Total (50)	130	90 (69.2)	63 (48.5)	13 (10.0)	64 (49.2)	1 (0.8)	0
Germany	Paediatrician (24)	70	17 (24.3)	56 (80.0)	4 (5.7)	47 (67.1)	4 (5.7)	0
	Neuropaediatrician (9)	26	6 (23.1)	25 (96.2)	1 (3.8)	22 (84.6)	0	0
	Psychiatrist (12)	35	8 (22.9)	31 (88.6)	1 (2.9)	28 (80.0)	0	0
	Paediatric/adolescent psychiatrist (7)	20	0	20 (100.0)	1 (5.0)	17 (85.0)	1 (5.0)	0
	Total (52)	151	31 (20.5)	132 (87.4)	7 (4.6)	114 (75.5)	5 (3.3)	0
Italy	Paediatrician (28)	56	41 (73.2)	20 (35.7)	11 (19.6)	18 (32.1)	3 (5.4)	0
	Neuropaediatrician (38)	74	50 (67.6)	29 (39.2)	18 (24.3)	21 (28.4)	5 (6.8)	0
	Neuropsychiatrist (8)	14	10 (71.4)	5 (35.7)	0	4 (28.6)	0	0
	Total (74)	144	101 (70.1)	54 (37.5)	29 (20.1)	43 (29.9)	8 (5.6)	0
Netherlands	Paediatrician (30)	37	29 (78.4)	2 (5.4)	0	9 (24.3)	1 (2.7)	0
	Neuropsychiatrist (1)	1	0	0	0	0	0	0
	Psychiatrist (9)	12	12 (100.0)	0	0	4 (33.3)	0	0
	Paediatric/adolescent psychiatrist (16)	24	19 (79.2)	1 (4.2)	2 (8.3)	9 (37.5)	0	0
	Total (56)	74	60 (81.1)	3 (4.1)	2 (2.7)	22 (29.7)	1 (1.4)	0
Spain	Paediatrician (21)	60	50 (83.3)	14 (23.3)	16 (26.7)	13 (21.7)	5 (8.3)	0
	Psychiatrist (15)	40	28 (70.0)	15 (37.5)	4 (10.0)	13 (32.5)	2 (5.0)	0
	Paediatric/adolescent psychiatrist (14)	34	28 (82.4)	15 (44.1)	3 (8.8)	15 (44.1)	3 (8.8)	0
	Total (50)	134	106 (79.1)	44 (32.8)	23 (17.2)	41 (30.6)	10 (7.5)	0
UK	Paediatrician (23)	57	20 (35.1)	11 (19.3)	0	42 (73.7)	3 (5.3)	20 (35.1)
	Neuropaediatrician (2)	6	3 (50.0)	6 (100.0)	0	6 (100.0)	0	0
	Psychiatrist (7)	18	7 (38.9)	11 (61.1)	4 (22.2)	6 (33.3)	0	9 (50.0)
	Paediatric/adolescent psychiatrist (26)	65	16 (24.6)	37 (56.9)	2 (3.1)	47 (72.3)	1 (1.5)	9 (13.8)
	Total (58)	146	46 (31.5)	65 (44.5)	6 (4.1)	101 (69.2)	4 (2.7)	38 (26.0)
Total EU		779	434 (55.7)	361 (46.3)	80 (10.3)	385 (49.4)	29 (3.7)	38 (4.9)

*n*  number of physician responses, *ADHD* attention deficit/hyperactivity disorder, *DSM-IV* Diagnostic and Statistical Manual of Mental Disorders, 4th edn, *ICD-9/ICD-10* International Classification of Diseases, Revision 9 or Revision 10, *SNAP-IV* Swanson, Nolan and Pelham Rating Scale, Version IV, *Connors* ADHD Connors Test, *IOWA* Inattention/Overactivity With Aggression screening tool

### Patient characteristics

#### Patient demographics

**Fig. 2:**
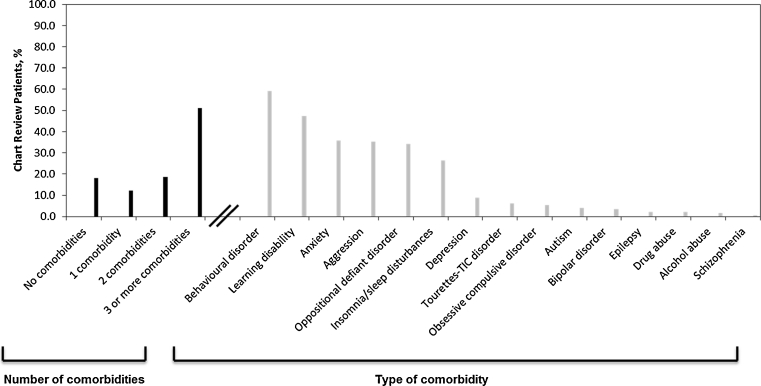
Number and type of comorbidities at time of ADHD diagnosis, all countries (*n* = 779). Note that physicians can select more than one comorbidity at diagnosis

Patient demographics are presented in Table 2. Patient mean (SD) age at diagnosis was 8.9 (2.6) years old, which was similar across countries. The mean (SD) patient age at the start of chart abstraction was 12.1 (2.6) years, with little variation across countries. The majority of the patients (77.5 % [604/779]) were male and Caucasian (85.8 % [557/649]). In France, the ethnicity of the patient was not allowed to be reported; therefore, the ethnicity data are based on a sample of 649 instead of 779 patients. Approximately 49 % (384/779) of patients had one to three comorbidities at the time of diagnosis (Fig. 2). The most commonly reported comorbidities were behavioural disorders (59.3 % [462/779]), learning disabilities (47.4 % [369/779]), anxiety (35.7 % [278/779]), aggression (35.3 % [275/779]) and oppositional defiant disorder (34.3 % [267/779]).

**Table 2:** Demographics for patients aged 17 years and younger

	France	Germany	Italy	Netherlands	Spain	UK	Total (Europe)
Number of patients	130	151	144	74	134	146	779
Gender, *n* (%)							
Male	111 (85.4)	113 (74.8)	108 (75.0)	57 (77.0)	97 (72.4)	118 (80.8)	604 (77.5)
Female	19 (14.6)	38 (25.2)	36 (25.0)	17 (23.0)	37 (27.6)	28 (19.2)	175 (22.5)
Age at ADHD diagnosis							
Mean (SD)	9.1 (2.5)	8.4 (2.1)	8.7 (2.9)	8.6 (2.6)	9.0 (2.3)	9.3 (2.8)	8.9 (2.6)
Median	9	8	8	9	9	9	9
Range (min, max)	3, 14	2, 15	4, 14	4, 15	3, 15	4, 15	2, 15
Age at chart abstraction							
Mean (SD)	12.4 (2.6)	11.8 (2.3)	11.8 (2.8)	11.7 (2.5)	12.1 (2.5)	12.7 (2.7)	12.1 (2.6)
Median	12	12	11	12	12	13	12
Range (min, max)	6, 17	6, 17	7, 17	7, 17	7, 17	6, 17	6, 17
Patient currently in school, *n* (%)							
Yes	109 (83.8)	144 (95.4)	135 (93.8)	72 (97.3)	123 (91.8)	126 (86.3)	709 (91.0)
No	15 (11.5)	5 (3.3)	9 (6.3)	2 (2.7)	10 (7.5)	16 (11.0)	57 (7.3)
Don't know	6 (4.6)	2 (1.3)	0 (0.0)	0 (0.0)	1 (0.7)	4 (2.7)	13 (1.7)
Private health insurance, *n* (%)							
Yes	85 (65.4)	22 (14.6)	8 (5.6)	74 (100)	20 (14.9)	4 (2.7)	213 (27.3)
No	24 (18.5)	129 (85.4)	99 (68.8)	0 (0.0)	90 (67.2)	136 (93.2)	478 (61.4)
Don't know	21 (16.2)	0 (0.0)	37 (25.7)	0 (0.0)	24 (17.9)	6 (4.1)	88 (11.3)

*SD* standard deviation, *min* minimum, *max* maximum, *ADHD* attention deficit/hyperactivity disorder

#### ADHD diagnosis

Feedback from teachers at school was the most common reason for seeking ADHD evaluations (78.3 % [610/779]) across all countries. Challenges raised by parents/family were also a common reason for seeking evaluation among 63.8 % (497/779).

Across all countries, the commonly used diagnostic criteria included the *Diagnostic and Statistical Manual of Mental Disorders,* 4th edition (DSM-IV) (55.7 % [434/779]), and *International Classification of Diseases, Revision 9* or *10* (ICD-9/ICD-10) (46.3 % [361/779]). ADHD Connors Test (49.4 % [385/779]) was also commonly used, though physicians may have used more than one set of diagnostic criteria to make an ADHD diagnosis. As presented in Table 1, within each country the diagnostic criteria utilized varied based on physician specialty.

#### ADHD symptoms and impairment levels at diagnosis

**Fig. 3:**
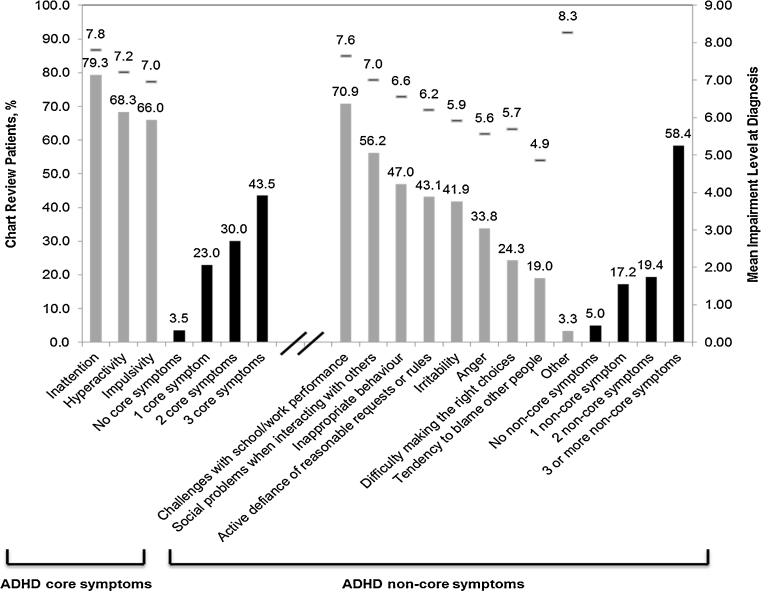
Symptoms and impairment levels at diagnosis, all countries (*n* = 779). Note that physicians can select more than one symptom or impairment at diagnosis. *Horizontal bars* and data reflect the mean level of impairment for that symptom at diagnosis

The most common symptoms at diagnosis were the core symptoms of ADHD (inattention, impulsivity and hyperactivity) as well as impairments with school performance (Fig. 3). Approximately 44 % (339/779) of patients were reported as having all three core symptoms at the time of diagnosis (Fig. 3). Furthermore, the majority of patients (63.7 % [496/779]) had three or more additional symptoms at diagnosis. More than a third of the patients exhibited anger, irritability and inappropriate behaviours. At the time of diagnosis, most physicians reported the level of impairment in inattention (67.9 % [529/779]), impulsivity (53.0 % [413/779]), hyperactivity (57.3 % [446/779]) and challenges in school (66.1 % [515/779]) as 8 or above on a ten-point scale. Most patients showed evidence of impairment both at school (91.1 % [710/779]) and at home (81.4 % [634/779]).

### ADHD treatment

#### ADHD treatment goals

**Fig. 4:**
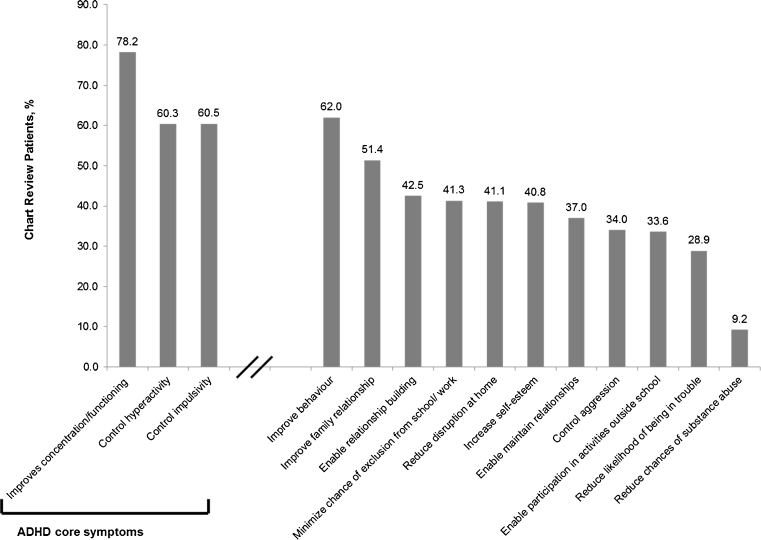
Therapeutic goals of ADHD treatment at the time of diagnosis for chart review patients (>1 %), all countries (*n* = 779). Note that physicians can select more than one therapeutic goal of ADHD treatment at time of diagnosis

At diagnosis, most physicians across all six countries indicated (based on the provided choices) that a goal of ADHD treatment was to improve attention and functioning at school or work for their patients (78.1 % [609/779]; Fig. 4). Additional therapeutic goals were strongly aligned with controlling the other core ADHD symptoms of hyperactivity (60.3 % [470/779]) and impulsivity (60.5 % [471/779]). Overall, the physicians stated that they wanted control of all three core symptoms for 38.1 % (297/779) of their patients. Apart from core symptom-related goals, the next most commonly indicated goals of treatment were to improve behaviour (62.0 % [483/779]), family relationships (51.4 % [400/779]) and relationship building (42.5 % [331/779]).

#### ADHD treatments utilized and treatment patterns

At the time of chart abstraction, 49.6 % (386/779) were on their first ADHD treatment regimen (either drug or nondrug), 37.9 % (295/779) had one previous line of therapy and the remaining 12.6 % (98/779) of patients received two or more previous lines of therapy (Table 3). Germany had the most number of patients with three or more total lines of therapy (19.9 % [29/151]). Approximately 80 % of patients had ≤0.5 years between ADHD diagnosis and the initiation of treatment.

**Table 3:** ADHD treatment utilization and patterns

	France		Germany		Italy		Netherlands		Spain		UK		Total (Europe)	
	*N*	%	*N*	%	*N*	%	*N*	%	*N*	%	*N*	%	*N*	%
Total number of patients (*N*)	130		151		144		74		134		146		779	
Lines of treatment														
One	85	65.4	77	51.0	81	56.3	46	62.2	69	51.5	64	43.8	386	49.6
Two	37	28.5	45	29.8	60	41.7	19	25.7	48	35.8	55	37.7	295	37.9
Three	7	5.4	24	15.9	3	2.1	7	9.5	15	11.2	17	11.6	73	9.4
Four	1	0.8	3	2.0	0	0.0	1	1.4	1	0.8	7	4.8	18	2.3
Five or more	0	0.0	2	1.3	0	0.0	1	1.4	1	0.8	3	2.1	7	0.90
Current treatment type^a^														
No treatment^b^	12	9.2	14	9.37	9	6.3	2	2.7	2	1.5	7	4.8	46	5.9
Pharmacotherapy only	71	54.2	84	55.6	33	22.9	45	60.8	48	35.8	93	63.7	374	48.0
BT only	10	7.7	10	6.6	59	41.0	1	1.4	9	6.7	1	0.9	90	11.6
Pharmacotherapy and BT	37	28.5	43	28.5	43	29.9	26	35.1	75	56.0	45	30.8	269	34.5
Current treatment class^c, d^														
Short-acting MPH	39	34.2	56	36.8	43	54.4	25	31.7	0	0.0	16	10.2	179	25.1
Long-acting MPH^e^	73	64.0	82	54.0	0	0.0	45	57.0	91	68.9	107	68.2	398	55.8
Short-acting AMP	0	0.0	1	0.76	7	8.9	1	1.3	0	0.0	2	1.3	11	1.5
Atomoxetine^f^	1	0.9	13	8.6	29	36.7	8	10.1	13	9.9	32	20.4	96	13.5
Others	1	0.9	0	0.0	0	0.0	0	0.0	28	21.2	0	0.0	29	4.1

*N* total number of patients, *BT* behavioural therapy, *MPH* methylphenidate, *AMP* amphetamine

^a^Percentages are based on the total number of patients reporting treatment type

^b^Not included in any analyses

^c^Percentages are based on the total number of patients reporting treatment class

^d^Treatment could be monotherapy or combination therapy

^e^Long-acting MPH is not approved for use in Italy

^f^Atomoxetine is not approved for use in France

Overall, 34.5 % (269/779) were treated with a combination of pharmacotherapy and BT, whereas 48.0 % (374/779) of patients were undergoing treatment with pharmacotherapy only and 11.6 % (90/779) with BT only (Table 3). A small percentage of patients (5.9 % [46/779]) did not receive any treatment at the time chart abstraction was completed.

Across all countries, excluding Italy where long-acting methylphenidates are not approved for use, long-acting methylphenidate was the predominant treatment choice (among 55.8 % of patients) either as monotherapy or in combination. Overall, short-acting methylphenidates (25.1 % of patients) and atomoxetine (13.5 % of patients) were the next most commonly used treatments (Table 3).

### Association between key predictor variables and outcomes

Overall, 71.4 % of patients with ADHD were estimated by physicians to have high rates of adherence (>80 % weekdays and >50 % weekends). Adherence was estimated to be highest in patients receiving combination medication therapy (80.0 %) and lowest for patients receiving BT only (12.2 %). However, according to physician judgement, only 30.8 % of patients showed ‘complete symptom control’. Symptom control was highest for patients receiving long-acting methylphenidate (36.2 %) and lowest for patients receiving BT only (15.6 %). Only 31.8 % of physicians were ‘very satisfied’ with their patients’ ADHD treatment at the time of chart abstraction. Highest physician satisfaction was reported for patients receiving medication combination therapy (38.8 %) and lowest satisfaction was reported for patients receiving BT only (14.4 %).

Generally, we found positive associations between the need to improve inattention as a treatment goal at diagnosis, increased patient engagement and increased family involvement and all three outcomes (adherence, symptom control and physician satisfaction with treatment). The odds of a favourable outcome for patients with improving inattention as a treatment goal were approximately twofold higher than for those patients for whom improving inattention was not a stated goal (OR = 2.1; 95 % CI = 1.5–3.1; *p* < 0.0001; Table 4). Similarly, improved mean scores for patient engagement and family involvement were positively associated with adherence, symptom control and physician satisfaction. For example, adherent patients had a mean engagement score of 6.7 versus 5.7 for nonadherent patients (Table 4).

**Table 4:** Significant associations between patient clinical characteristics and binary outcomes (adherence, symptom control and treatment satisfaction)

Categorical variables	Adherence, *N* = 710 (71.4 %)		Complete symptom control, *N* = 730 (30.8 %)		Physician very satisfied, *N* = 730 (31.8 %)		Continuous variables	Means for adherent vs nonadherent patients	Means for complete symptom control vs no complete symptom control patients	Means for patients with very satisfied physicians vs not very satisfied physicians
	Odds ratio	95 % CI	Odds ratio	95 % CI	Odds ratio	95 % CI				
Treatment goal at diagnosis							Comorbidities at diagnosis			
Improve inattention	2.1^a^	1.5–3.1	2.5^a^	1.6–3.9	3.0^a^	1.9–4.7	Number of comorbidities	—	2.2 vs 3.0^c^	2.2 vs 3.0^b^
Predominant symptoms at diagnosis							Impairment at diagnosis			
Anger	–	–	–	–	0.6^b^	0.4–0.8	Impulsivity	–	6.5 vs 7.2^b^	–
Comorbidities at diagnosis							Irritability	–	5.4 vs 6.2^c^	5.4 vs 6.2^b^
Aggression	–	–	0.5^b^	0.4–0.7	–	–	Anger	–	4.8 vs 5.9^c^	4.9 vs 5.9^c^
Tourette syndrome	–	–	0.2^c^	0.1–0.5	0.3^b^	0.1–0.6	Active defiance	–	5.6 vs 6.5^c^	5.5 vs 6.5^c^
Learning disability	–	–	–	–	0.6^b^	0.4–0.8	Inappropriate behaviour	–	6.0 vs 6.9^c^	5.9 vs 6.9^c^
Depression	0.3^c^	0.2–0.5	–	–	–	–	Problems with social interactions	–	6.4 vs 7.3^c^	6.4 vs 7.3^c^
Bipolar	0.2^b^	0.1–0.6	–	–	–	–	Tendency to blame others	4.6 vs 5.4^c^	4.4 vs 5.1^b^	–
							Patient engagement	6.7 vs 5.7^a^	7.3 vs 5.9^a^	7.4 vs 5.9^a^
							Family involvement	8.0 vs 7.1^a^	8.5 vs 7.5^a^	8.5 vs 7.4^a^

Odds ratios were calculated for dichotomous characteristics. Odds ratios consist of the odds of each favourable outcome (e.g. adherence) for the indicated row variable (e.g. patients with ‘improve inattention’ as treatment goal) over the odds of the favourable outcome in the complementary group of patients for the row variable. X vs Y values denote mean values for continuous characteristics. For each outcome, the proportion of patients with a favourable outcome is indicated in parenthesis on the column headings

*–* association was not significant at *p* < 0.001, *N* number of patients with available data on outcome

^a^Denotes a significant positive association, *p* < 0.0001

^b^Denotes a significant negative association, 0.0001 ≤ *p* < 0.001

^c^Denotes a significant negative association, *p* < 0.0001

Alternatively, increased comorbidities at diagnosis (both total number and specific types) were negatively associated with outcomes. For instance, in patients with comorbid Tourette syndrome, the odds of favourable symptom control and physician satisfaction were reduced fivefold (OR = 0.2; 95 % CI = 0.1–0.5; *p* < 0.0001) and 3.3-fold (OR = 0.3; 95 % CI = 0.1–0.6; 0.0001 ≤ *p* < 0.001), respectively. Patients perceived to have complete symptom control had a mean number of 2.2 comorbidities compared to a mean number of 3.0 comorbidities in those patients without complete symptom control (*p* < 0.0001; Table 4).

The presence of certain ADHD symptoms at the time of diagnosis was also associated with unfavourable outcomes. The odds of favourable physician satisfaction with ADHD treatment was reduced 1.7-fold in patients with the symptom of anger at the time of diagnosis (OR = 0.6; 95 % CI = 0.4–0.8; 0.0001 ≤ *p* < 0.001). Furthermore, as seen in Table 4, there were multiple impairments at the time of diagnosis that were negatively associated with adherence, symptom control and physician satisfaction. For example, patients whose symptoms were completely controlled demonstrated a mean ‘active defiance’ impairment score of 5.6 compared with a score of 6.5 in those who did not demonstrate complete symptom control (*p* < 0.0001).

### Country differences

Patient comorbidities at the time of ADHD diagnosis varied considerably across countries. The majority of countries reported at least one patient with each comorbidity represented in Fig. 2; however, data from Germany did not include any patients with schizophrenia, obsessive compulsive disorder, alcohol abuse, epilepsy or drug abuse; the Netherlands did not report any patients with bipolar disorder, schizophrenia or alcohol abuse; and Spain and the UK did not report any patients with schizophrenia. Also, patient membership to private insurance differed based on country. One hundred percent of patients from the Netherlands had private insurance compared with just 2.7 % of patients from the UK.

The diagnostic criteria used by the treating physicians varied based on country. The DSM-IV criteria for diagnosing ADHD were most commonly used in France (69.2 % [90/130]), Italy (70.1 % [101/144]), the Netherlands (81.1 % [60/74]) and Spain (79.1 % [106/134]). ADHD Connors Test was frequently part of the diagnostic assessment in the UK (69.2 % [101/146]). ICD-9/ICD-10 diagnostic criteria were most commonly used in Germany (87.4 % [132/151]).

Country level variation in the treatment of ADHD was also noted. The UK had the highest rate of treatment with pharmacotherapy alone at 63.7 % (93/146). Treatment with BT alone was most common in Italy (41 % [59/144]) and least common in the UK (<1 % [1/146]). More than 54 % of patients in Italy used short-acting methylphenidates, while Spain did not report any use at all. Atomoxetine was most commonly used in Italy (36.7 %), but it was not available in France because it has not been approved by the Comité Economique des Produits de Santé. Short-acting amphetamines were not used in France or Spain and were used by less than 2 % of patients in Germany, the UK and the Netherlands. The highest use for short-acting amphetamines is in Italy (9 %) (Table 3).

The percentage of patients who adhered to their ADHD treatment ranged from 50.8 % (62/122) in Italy to 80.3 % (94/117) in France. Physicians from the Netherlands reported the highest percentages of ‘complete symptom control’ (52.8 % [38/72]), while Italian physicians reported the lowest percentage (16.4 % [22/134]). Methylphenidate ‘very good responders’ ranged from 11.1 % (16/144) in Italy to 35.1 % (53/151) in Germany. Physicians in Germany (46.0 % [63/137]) and the Netherlands (59.7 % [43/72]) reported the highest percentages of ‘very satisfied’, while physicians in Italy reported the lowest percentage (14.8 % [20/135]).

## Discussion

Country-level variations were evident in patient characteristics as well as ADHD diagnosis, treatment and outcomes. Patients had differences in comorbidities at the time of ADHD diagnosis across countries. Therefore, it is important that all treating physicians have a similar knowledge and understanding not only of the diagnosis and management of ADHD but also the associated comorbid conditions to deliver optimal treatment based on individual patient needs and a global view of the problems. Evaluations of differences in treatment based on patient comorbidities are planned for future analyses.

Patients also had differences in private insurance membership based on country. In the Netherlands, all patients were members of private insurance, but in the UK, where ADHD treatment is free under the National Health Service, only 2.7 % had private insurance. The discrepancies in membership to private insurance across countries may influence ADHD management and treatment decisions for patients. Future analyses are planned to better understand ADHD management differences within countries and specialties as well as differences in treatment based on comorbidities.

Within each country, the criteria used to diagnose ADHD varied by physician specialty as well. The criteria provided in DSM-IV were the most commonly used to diagnose ADHD in the countries studied. Use of the Swanson, Nolan and Pelham IV Rating Scale (SNAP) and the Inattention/Overactivity With Aggression (IOWA) screening tool increased each year from 2004 to 2007. Although scales like SNAP and IOWA are valuable for screening patients for ADHD, if used alone, they may lead to overdiagnosis or misdiagnosis. Adoption of standardized diagnostic criteria across countries may address some of the variability noted worldwide in ADHD prevalence [15, 19]. ADHD diagnosis and management differences within countries and within physician specialties are planned for future analyses.

At ADHD diagnosis, physicians regarded core ADHD symptom control as a primary therapeutic goal. In the short term, controlling core symptoms is of key importance as hyperactivity, impulsivity and inattention frequently create dysfunction for patients, their families and their peers. However, it is important to note that ADHD treatment is not limited to symptom control only. Treatment goals must also include longer-term objectives such as improving the quality of life for both the patient and their families and reducing functional impairment for the patient [18].

In this study of ADHD treatment in European countries, more than half of patients received long-acting methylphenidate as the treatment of choice, except in Italy where it was not approved and therefore short-acting methylphenidates were the treatment of choice. This may also account for a higher rate of BT only and a lower rate of treatment response and treatment satisfaction in Italy compared with other countries. Whether or not BT was available through national health plans and consideration of waiting times may have affected these numbers. In addition, differing recommendations from the various physician specialties and other variations in availability of medication may have also influenced these data. The rationale behind the choices of ADHD treatments (i.e. symptom severity, comorbidity, etc.) is planned for further analysis.

Overall, across all of the countries studied, patients with ADHD were considered by physicians to have high rates of adherence (>80 % weekdays, >50 % weekends) with treatment on school or working days. However, less than one third of the patients were perceived by their physicians to exhibit ‘complete symptom control’ and less than one third of physicians were ‘very satisfied’ with the current ADHD treatment for their patients. The low overall percentages and high country variability for ‘complete symptom control’ and ‘very satisfied’ may be indicative of cultural differences among countries in the acceptability of physicians indicating dissatisfaction with ADHD therapy or as a result of inconsistency among countries in shared decision-making between physicians and patients/caregivers. We observed that patient/family involvement in the disorder and its treatment was positively associated with adherence, symptom control and physician satisfaction with treatment, reflecting the influence on treatment outcomes of shared decision-making [25]. In addition, low symptom control and satisfaction may be influenced by the lack of optimal treatment. In this study, whether or not titration of medication was optimal was not possible to ascertain. Clearly, physicians should endeavour to optimize management with pharmacological and nonpharmacological treatments currently available. Treatment should be individualized for each patient to ensure optimal response. However, this lack of symptom control and physician satisfaction with currently available treatment suggests that there also remains an unmet need in the six European countries studied with regard to available ADHD treatment options. Patients may be partial responders or nonresponders to one medication for ADHD, but that is not predictive of response to another class of medications [17, 18, 20]. Therefore, treatment optimization may include consideration of other ADHD medication classes not discussed herein, such as long-acting amphetamines [6, 26], alpha-2 agonists [7, 11, 16, 21] or ongoing BT. Management of coexisting conditions is also vital to improve overall outcomes, which was not possible to assess in this study.

### Limitations

This study was a retrospective chart review with inherent study design limitations, including the potential for incomplete or missing documentation as well as the inability to establish causality. The potential for selection bias in this study should be considered due to the convenience sampling strategy implemented. It is not known whether a greater number of physicians participated from secondary referral centres compared with those from primary care settings; thus, it is not possible to determine whether an oversampling of charts from more complex or difficult patients occurred in this study. Confounding factors included the variable availability of ADHD treatments, including BT, across countries and the lack of information regarding whether a patient started initial ADHD therapy in a clinic that was different from the clinic where the actual medical record abstraction occurred. Because this was an observational study of a convenience sample where patient charts were not sampled randomly, the generalizability of these study results to the entire European population may be limited. Furthermore, this study was conducted for descriptive and exploratory purposes and was not designed for testing of particular statistical hypotheses. As such, *p*-values presented in the results should be considered descriptive. However, given the fact that this is the first work that attempted to study real-world practice variation and management of ADHD across European countries, the evidence generated in this research still provides valuable additional information to the body of literature in ADHD.

## Conclusions

ADHD management differs across European countries and across physician specialties. Variations in patient characteristics as well as ADHD diagnosis, treatment and outcomes were evident between countries. Although a high proportion of patients were reported to be adherent to currently available therapy in Europe, less than one third of physicians perceived patients as having complete ADHD symptom control and less than one third of physicians were satisfied with ADHD treatments for their patients. These results suggest that European patients may benefit from better standardization of ADHD management across countries and additional treatment options.

## Appendix

Web-based chart abstraction form: key questions

Which of the following statements best describes the level of compliance of this patient with this treatment for each specific circumstance?

*Single answer per column*

**Table:**

	School/working days	Weekends	Holidays
The patient follows the treatment prescribed more than 80 % of the time	1	1	1
The patient follows the treatment prescribed between 51–80 % of the time	2	2	2
The patient follows the treatment prescribed between 25–50 % of the time	3	3	3
The patient follows the treatment prescribed less than 25 % of the time	4	4	4
Not applicable / the treatment is not prescribed for this circumstance	5	5	5
Don’t know	99	99	99

How well controlled are ADHD symptoms for this patient with this current treatment?

*Single answer*

**Table:**

Completely controlled	1
Moderately controlled	2
Poorly controlled	3
Not controlled at all	4
Don’t know	99

How would you define your level of overall satisfaction with this current treatment?

*Single answer*

**Table:**

Very satisfied	1
Moderately satisfied	2
Neither satisfied or dissatisfied	3
Moderately dissatisfied	4
Very dissatisfied	5

## Abbreviations

ADHD: Attention deficit/hyperactivity disorder
BT: Behavioural therapy
Connors: ADHD Connors Test
DSM-IV: Diagnostic and Statistical Manual of Mental Disorders 4th edn
ICD-9/ICD-10: International Classification of Diseases 9th and 10th Revision
IOWA: Inattention/Overactivity With Aggression screening tool
SNAP-IV: Swanson, Nolan and Pelham IV Rating Scale
